# The price of conserving avian phylogenetic diversity: a global prioritization approach

**DOI:** 10.1098/rstb.2014.0004

**Published:** 2015-02-19

**Authors:** Laura A. Nunes, Samuel T. Turvey, James Rosindell

**Affiliations:** 1 Centre for Biodiversity and Environment Research, University College London, London WC1E 6BT, UK; 2 Institute of Zoology, Zoological Society of London, Regent's Park, London NW1 4RY, UK; 3 Imperial College London, Silwood Park Campus, Ascot, Berkshire SL5 7PY, UK

**Keywords:** conservation prioritization, phylogeny-based conservation, Noah's Ark problem, EDGE

## Abstract

The combination of rapid biodiversity loss and limited funds available for conservation represents a major global concern. While there are many approaches for conservation prioritization, few are framed as financial optimization problems. We use recently published avian data to conduct a global analysis of the financial resources required to conserve different quantities of phylogenetic diversity (PD). We introduce a new prioritization metric (ADEPD) that After Downlisting a species gives the Expected Phylogenetic Diversity at some future time. Unlike other metrics, ADEPD considers the benefits to future PD associated with downlisting a species (e.g. moving from Endangered to Vulnerable in the International Union for Conservation of Nature Red List). Combining ADEPD scores with data on the financial cost of downlisting different species provides a cost–benefit prioritization approach for conservation. We find that under worst-case spending $3915 can save 1 year of PD, while under optimal spending $1 can preserve over 16.7 years of PD. We find that current conservation spending patterns are only expected to preserve one quarter of the PD that optimal spending could achieve with the same total budget. Maximizing PD is only one approach within the wider goal of biodiversity conservation, but our analysis highlights more generally the danger involved in uninformed spending of limited resources.

## 1. Introduction

Conservation researchers and practitioners are widely aware that efforts to combat the current global biodiversity crisis and conserve threatened species are limited in practical terms by major financial constraints [1]. For example, resources currently allocated to bird conservation are unlikely to be enough to meet global biodiversity conservation targets in the next decade [2]. It is therefore essential to allocate scarce conservation resources in an efficient manner to maximize the preservation of biodiversity [3]. The barrier to resource availability has led to development of various different approaches for prioritizing conservation activities towards components of biodiversity that are considered to be most ‘important’ to protect [1]. These attempts to identify the best combination of species to save within a limited budget are possible solutions to what is known as the Noah's Ark problem [4].

Phylogenetic diversity (PD) is a key component of biodiversity and can be interpreted as a compound measure of all forms of genotypic, phenotypic and functional diversity [5,6]. PD was first proposed as a potential prioritization metric for biodiversity conservation over two decades ago [6,7], at a time when the field of phylogenetic tree building, necessary for calculating PD, was in its infancy. If phylogenetic trees are scaled in units of time (e.g. millions of years), this can provide a comparative metric for calculating the contribution of different species to the wider PD of the clade under consideration [7]. Conservation prioritization using PD has been demonstrated to constitute an effective approach for capturing the range of morphological and ecological diversity that has evolved in a given clade [8,9].

Several scoring methods have been proposed by different authors for calculating the contribution to global PD made by different species, most of which represent theoretical suggestions for PD-based conservation prioritization. One such method, ‘evolutionarily distinct and globally endangered’ (EDGE), has been adopted by the Zoological Society of London as a practical tool for prioritizing species-focused conservation programmes at a global level [10]. EDGE ranks species according to a combination of their level of evolutionary distinctiveness (ED), a measure of PD also known as fair proportion, and their International Union for Conservation of Nature (IUCN) Red List status (‘Global Endangerment’ or GE) [10]. In recent years, priority lists for the world's mammals [10], amphibians [11], corals [12] and birds [13] have been constructed based on the EDGE approach. EDGE and several other methods assign scores to species that are independent of the conservation status of other related taxa. An alternative approach, the HEDGE method [14], uses a Heightened-ED (HED) measure that also takes the conservation status of related species into account. In addition, HEDGE measures the change in the total expected PD at a given point in the future, when a focal species becomes completely safe through conservation action (i.e. its probability of extinction is reduced to 0) [15,16]. HEDGE thus ranks species according to their expected contribution to future PD [16], providing a different approach to prioritization compared to EDGE [17].

The EDGE and HEDGE approaches provide a variety of methods for incorporating PD into conservation priority-setting at an international level [18]. In real terms, however, there is still a long way to go for effective conservation of PD. Among the vertebrate groups so far assessed using the EDGE approach, 50% of the top 100 birds, 66% of the top 100 mammals and 85% of the top 100 amphibians are receiving little or no conservation attention in terms of either targeted research or practical recovery actions (http://edgeofexistence.org (accessed on 1 May 2014)), and the great majority of conservation resources are still allocated towards a small subset of large-bodied charismatic species (e.g. artiodactyls, carnivores, perissodactyls and primates within mammals) that typically do not represent a significant level of PD within their wider clades [19,20]. Species that represent disproportionately high levels of PD are also being lost, as demonstrated by the recent extinction of the Yangtze River dolphin or Baiji (*Lipotes vexillifer*), which was identified as the global top-priority mammal species in the first iteration of the EDGE mammal list [10,21].

The effectiveness of conservation actions, both for preserving PD and more generally, is dependent upon efficient allocation of limited global resources available to international conservation practitioners [22]. Efficiency and success of conservation programmes will be increased by financial complementarity to conservation practice, for example, if different organizations are able to share knowledge and resources rather than duplicating efforts for some priority areas and neglecting others through a non-coordinated approach. Within a phylogeny-based prioritization context, optimal allocation of resources is best determined by combining a measure of phylogenetic benefit, probability of extinction and the associated conservation costs necessary to decrease this extinction probability [23]. Optimal resource allocation may also be determined by a return-on-investment approach that demonstrates the increase in PD being preserved per unit cost required for different conservation activities [24]. Several studies have successfully resolved the Noah's Ark problem computationally using simulated balanced phylogenetic trees and unit costs [25–27]. However, these theoretical solutions have rarely been implemented in a real-world conservation context (but see [26,27] for examples), and real phylogenies tend to be unbalanced due to contingent factors driving regional and taxon-specific variation in diversification, extinction and historical biogeography [28,29].

Comparative data on the predicted costs associated with conservation recovery of different species to meet biodiversity targets are not available across most higher-order clades. The costs necessary to reduce extinction risk by at least one Red List category within a 10 year timeframe have, however, recently been estimated on the basis of relevant expert knowledge for a cohort of 210 globally threatened (Vulnerable, Endangered and Critically Endangered) bird species, constituting 19% of all threatened birds [2], for which comparative phylogenetic data are also now available [13,30]. Here we use these independent sets of data to determine an explicit return-on-investment to maximize the cost-effective conservation of PD. To this end, we introduce a conservation prioritization method named ‘after downlisting expected phylogenetic diversity’ (ADEPD), which represents a measure of the change in the total expected future PD after a species is downlisted by one IUCN Red List category (e.g. from Critically Endangered to Endangered, or Endangered to Vulnerable [16]). Downlisting species represents a conservation step that is expected to reverse current global biodiversity trends by improving the conservation status of threatened species [31], and that constitutes one of the Aichi biodiversity targets for 2020 [32]. The ADEPD approach provides a means to combine available financial, extinction risk and phylogenetic datasets, comprising a novel approach to resolve the Noah's Ark problem if PD is considered to represent a primary basis for conservation prioritization. Although our current study investigates the cohort of threatened bird species for which sufficient data are currently available, our methods could be applied to assist with the cost-effective prioritization of wider species groups in the future as more extensive datasets become available to inform conservation management.

## 2. Material and methods

### (a) Data sources

We conducted our analysis on a distribution of 10 000 avian phylogenies composed of 9993 extant species with no unresolved polytomies following references [13,33]. We chose trees using the latest Parrott backbone available from birdtree.org [13,33]. These 10 000 trees enabled our results to account for any uncertainty in the assumed phylogenetic relationships. Extinction risk data for all bird species were obtained from the IUCN Red List [34] and transformed to probabilities of extinction (table 1). Species categorized as Data Deficient or Not Evaluated were assigned the same extinction risk as the Near Threatened category, as previous studies suggest that species in these categories are most likely to be reassessed as Near Threatened in the future [35]. Species categorized as Extinct in the Wild were given an extinction risk of 1, as they are considered unlikely to be reintroduced to the wild within 10 years [2].

**Table 1.** The number of species categorized in each IUCN threat category and the transformations of probabilities of extinction used in this study. (GE is the transformation used in traditional EDGE protocols while IUCN 50 is that used by our study. Data on transformations from Mooers *et al.* [18].)

IUCN category	no. species	GE	IUCN 50 years	IUCN 500 years	pessimistic scenario
Least Concern	7656	0	0.00005	0.0005	0.2
Near Threatened	877	1	0.004	0.02	0.4
Data Deficient	59	1	0.004	0.02	0.4
Not Evaluated	111	1	0.004	0.02	0.4
Vulnerable	710	2	0.05	0.39	0.8
Endangered	393	3	0.42	0.996	0.9
Critically Endangered	183	4	0.97	1	0.99
Extinct in the Wild	4	6	1	1	1

Different methods for transforming Red List categories into probabilities of extinction are available in the literature [15], with variation in estimated probability of extinction for a given threat category associated with factors such as relative difference in risk between categories, and time in the future (time window) to which the extinction probability is referring. The choice of which transformation to use for a particular analysis should therefore be based on the intentions and duration of planned conservation activities. For example, the pessimistic and IUCN 500 transformations (table 1) may be unlikely to prioritize Critically Endangered species, because downlisting such species by one threat category barely reduces the probability of extinction under the assumptions of these transformations. Our study aims to produce a balanced view of conservation prioritization, and so we chose the IUCN 50 year transformation over the more extreme IUCN 500 year or pessimistic scenarios and the less informative scenario of GE.

### (b) Analysis

For a phylogenetic tree *T* with *n* tips (species), each tip *i* is associated with IUCN Red List status information given by *R_i_* and a connecting edge of length given by *L_i_* for 1 ≤ *i* ≤ *n*. We use trees without polytomies and without a stem edge, so *T* will have exactly *n* − 2 further interior connecting edges with lengths given by *L_i_*, where *n* < *i* ≤ 2*n* − 2. The total phylogenetic diversity PD(*T*) of the tree *T* was measured by adding all of the edge lengths of the phylogenetic tree extracted using the *clade.matrix* function in the *caper* package [36]:
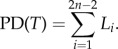
The probability of extinction *p*(*i*) of a tip of the tree *i* was given by the IUCN 50 transformation as defined in table 1 and the Red List status for that tip *R_i_* where 1 ≤ *i* ≤ *n.* The probability of extinction of an interior edge *p*(*i*), where *n* < *i* ≤ 2*n* − 2, was calculated as the product of the probabilities of extinction of its two daughter edges *p*(*j*) and *p*(*k*):
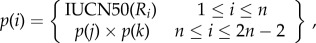
where edge *i* is connected to daughter edges *j* and *k*.

The expected phylogenetic diversity (EPD) of tree *T*, EPD(*T*), 50 years in the future assuming the IUCN 50 probabilities of extinction is therefore given by
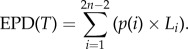
The ADEPD score of a tip *j* within a tree *T* for 1 ≤ *j* ≤ *n* is defined as the expected gain in future expected PD after a species is downlisted by one IUCN threat category:
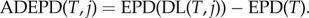


DL(*T*, *j*) represents the tree *T* after species *j* has been downlisted.

From the initial cohort of 210 globally threatened bird species for which relevant expert knowledge is available to estimate the costs of downlisting by one threat category within a 10 year timeframe [2], four species were excluded from analysis. The Tablas drongo (*Dicrurus menagei*), Príncipe thrush (*Turdus xanthorhynchus*) and Bahama oriole (*Icterus northropi*) were excluded as they are recent taxonomic splits not represented in the available phylogenetic tree template [30]; the golden parakeet (*Guaruba guarouba*) was also excluded as this species has recently been downlisted, and so its previously estimated downlisting cost is unlikely to still apply [34]. The remaining 206 species (102 Critically Endangered, 65 Endangered and 39 Vulnerable species) were downlisted in each of the 10 000 phylogenetic templates to obtain a distribution of the estimated benefit to EPD when downlisted.

For each of the 10 000 trees, we assigned ADEPD scores to each species methodically as described in the equations above. For each species, we then calculated the mean ADEPD score across all 10 000 trees. Next, we divided this by the associated costs of downlisting to establish a new measure for conservation prioritization: the EPD gain per unit cost (ADEPD-cost) score. We tested the correlation between ADEPD score, EDGE score and ADEPD-cost score using Pearson correlation coefficients.

In order to determine optimal sets of more than one species for downlisting, we needed to take into account that downlisting a species will change the ADEPD-cost scores for all the remaining species, an effect known as complementarity [16]. To account for complementarity we proceeded, for each tree, as follows: the species with the highest ADEPD-cost score for that tree was downlisted; the ADEPD-cost scores were then recalculated for the remaining species as these might have changed; we then downlisted the next best species based on the revised ADEPD-cost scores. We repeated the process for sets containing increasing numbers of species to be downlisted, right up to the limiting case where all species were included. The ADEPD-cost scores taking into account complementarity for each species across the 10 000 trees were then averaged, and species were ranked according to their averaged score to obtain an overall optimal prioritization ranking for sets of species in this cohort across all 10 000 trees. The 206 species were then downlisted sequentially based on their mean ADEPD scores taking into account complementarity. We calculated the cumulative funds spent and the mean quantity of PD gain (with standard deviation) across all 10 000 trees for each set of species. The result was a curve showing the mean outcome of spending different quantities of funding optimally. We also found the rate of change of this curve by numerical differential.

We used data on conservation expenditure for the cohort of 206 species over the past decade [2] to explore the estimated future gain in EPD if this budget was maintained for the next 10 years by multiplying the associated past species-specific expenditure with its ADEPD-cost score for each tree, assuming a linear response of EPD gain to unit cost spent. This provided an average amount of PD gain (with standard deviation) as a function of funds spent assuming current spending patterns. Less than 3% of species were reported to have received no conservation funding over the last decade; the black-cheeked ant-tanager (*Habia atrimaxillaris*) had no available estimates for recent expenditure on its conservation [2], and so we also assumed no resources were spent on this species during this time interval. We plotted the accumulated EPD gain against the accumulated costs of the conservation work. All modelling was scripted in R v. 3.0.0 [37], but using existing R functions and packages where stated [36].

## 3. Results

### (a) Characterizing and comparing the ADEPD ranking

**Figure 1.**
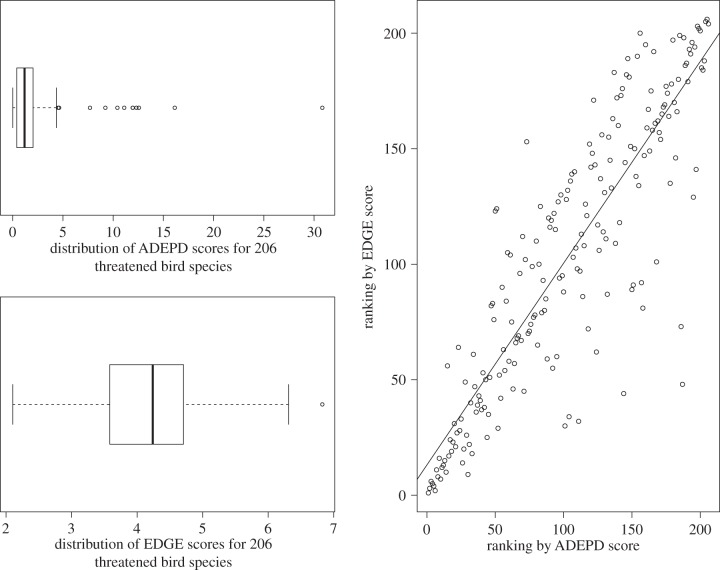
Distribution of ADEPD and EDGE scores as box plots and the correlation between ADEPD and EDGE rankings. The correlation between ADEPD and EDGE ranks for the top 20 species was *r* = 0.870 (*p* < 0.001) and for all 206 species was *r* = 0.698 (*p* < 0.001). Data on EDGE scores from Jetz *et al.* [16].

The ADEPD scores across all 206 species ranged between 0.004 and 30.819, but had a rather skewed distribution, with half of the species having a score between 0.408 and 2.019 (see the electronic supplementary material for the full dataset). The median ADEPD score was 1.179 (figure 1). The top-ranked ADEPD species was the giant ibis (*Thaumatibis gigantea*), which was also the top EDGE species (table 2). There was significant congruence between EDGE and ADEPD both for the top 20 species (*p* < 0.001) and overall (*p* < 0.001) based on our ADEPD scores and published EDGE scores (figure 1), demonstrating significant congruence between the two prioritization protocols. Despite this congruence, we found that certain species received significantly higher prioritization in one ranking than in the other. For example, the sister species pair composed of the Endangered thick-billed parrot (*Rhynchopsitta pachyrhyncha*) and the Endangered maroon-fronted parrot (*Rhynchopsitta terrisi*) were ranked 50 and 51, respectively, in the ADEPD ranking, but 123 and 124, respectively, in the EDGE ranking, which was unable to incorporate the additional risk of extinction for interior branches of the phylogenetic tree associated with having two closely related species that are both Endangered. Similarly, the Critically Endangered Madagascar fish-eagle (*Haliaeetus vociferoides*) was ranked higher in EDGE (32) than in ADEPD (111), because this species has close relatives that have a much lower risk of extinction. These differences arise because ADEPD gives greater priority to groups of closely related threatened species, such as the *Rhynchopsitta* sister species, whereas EDGE gives greater priority to highly threatened species irrespective of the threat status of their close relatives.

**Table 2.** Variation in the 20 species with highest ADEPD-cost score out of 206 globally threatened birds, not taking into account complementarity. (Data on expenditure from McCarthy *et al.* [2], phylogenetic trees from Jetz *et al.* [16,33] and conservation status from the IUCN Red List [37].)

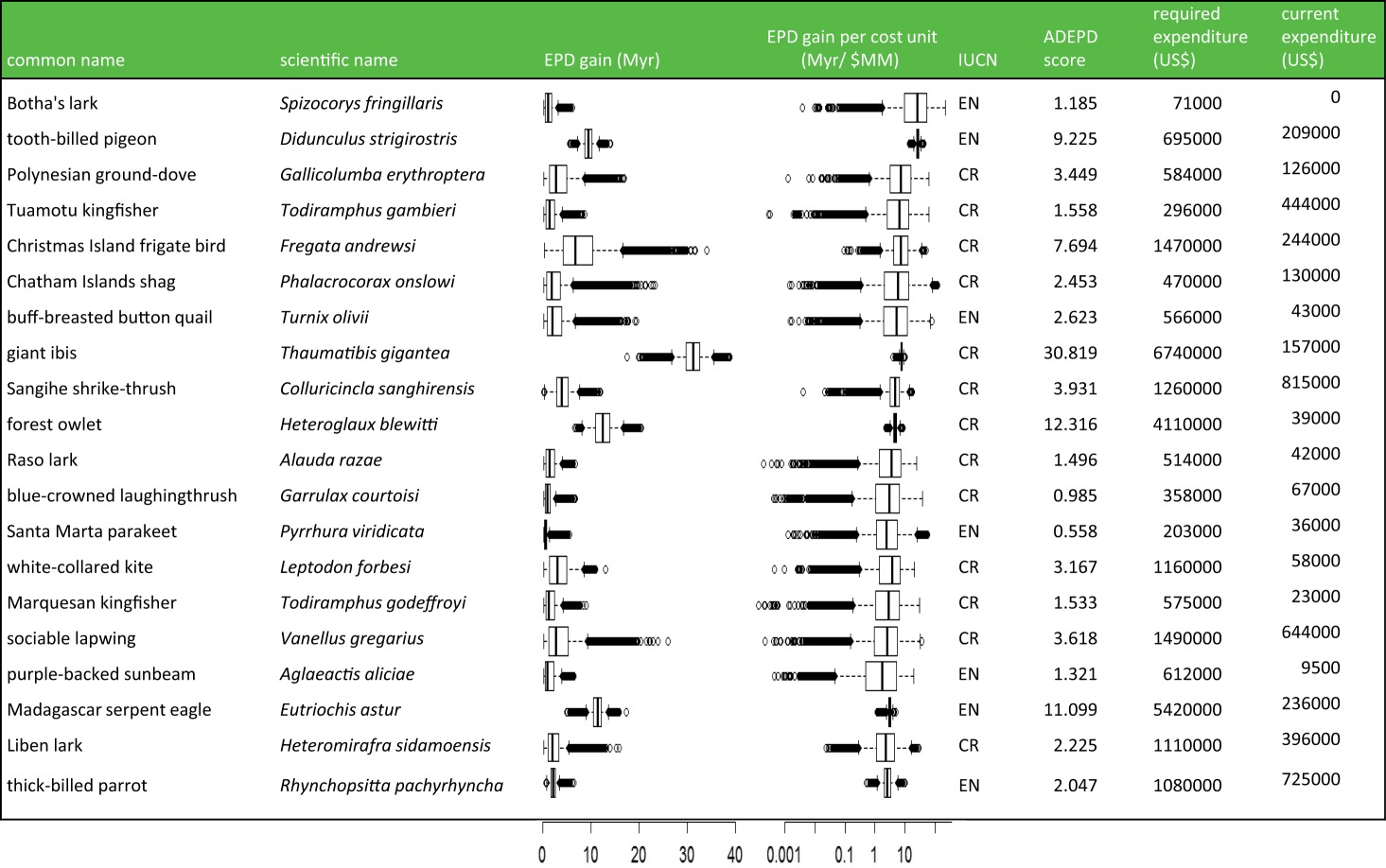

### (b) Financial analysis and the Noah's Ark problem

**Figure 2.**
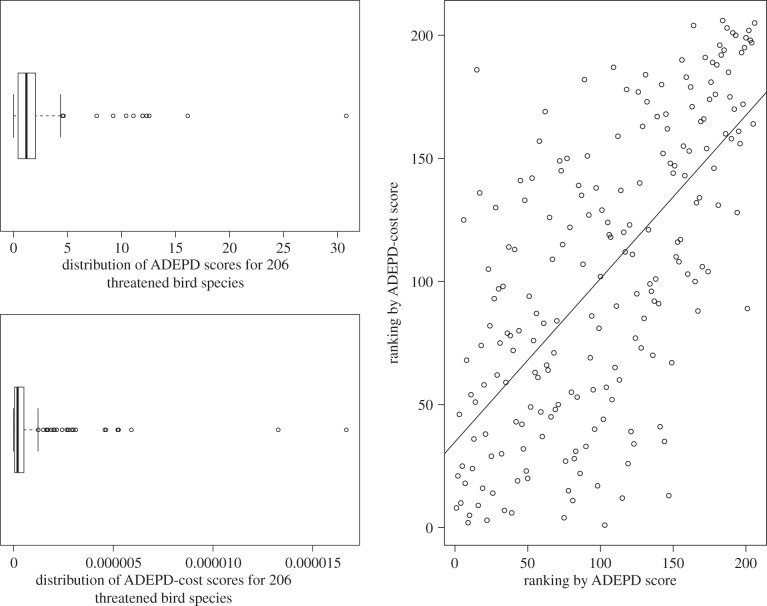
Distribution of ADEPD-cost scores and correlation between ranking from ADEPD score and ranking from ADEPD-cost score. There is a non-significant correlation between ADEPD and ADEPD-cost rankings for the top 20 species (*r* = 0.243, *p* = 0.301), but a significant correlation for all 206 species (*r* = 0.321, *p* < 0.001).

The ADEPD-cost scores across all 206 species ranged between 2.554 × 10^−9^ and 1.669 × 10^−5^, but, like the ADEPD scores, had a rather skewed distribution, with half of the species having a score between 4.586 × 10^−7^ and 5.1 × 10^−6^. The median ADEPD-cost score was 1.99 × 10^−6^ (figure 2). The top ADEPD-cost species was Botha's lark (*Spizocorys fringillaris*), with an estimated 16.69 years of EPD gain per dollar spent (table 2). The lowest ADEPD-cost species was the white-bearded antshrike (*Biatas nigropectus*), with 1 year of EPD gain costing $3915. There was no significant congruence between ADEPD and ADEPD-cost for the top 20 species (*p* = 0.301), showing significant differences in priority-setting between these different protocols (figure 2). A significant correlation was found when all 206 species were included in the test (*p* < 0.001). The differences between the rankings of two Critically Endangered species, the California condor (*Gymnogyps californianus*) (ranked number 6 in ADEPD and 125 in ADEPD-cost) and the blue-crowned laughingthrush (*Garrulax courtoisi*) (ranked number 115 in ADEPD and 12 in ADEPD-cost), are examples of the non-congruence between the two rankings, given that the costs of downlisting the laughingthrush were smaller by three orders of magnitude than those of the condor despite the benefit to EPD from downlisting the laughingthrush being only one order of magnitude smaller in comparison.

**Figure 3.**
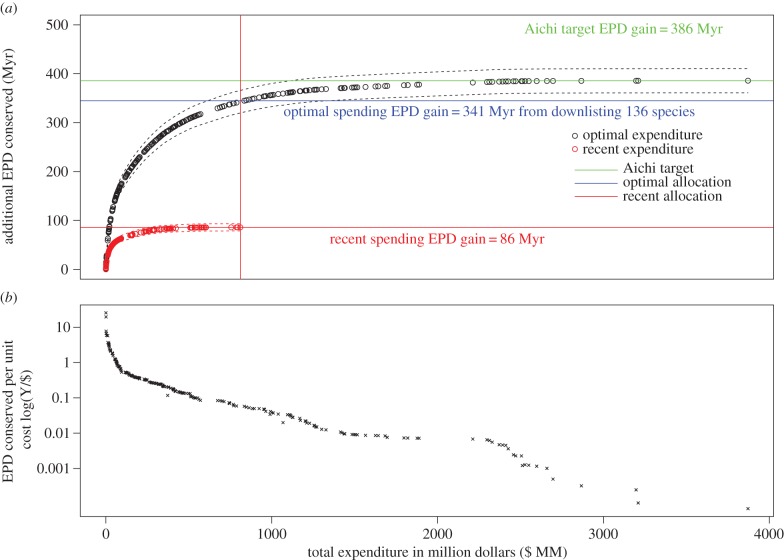
(*a*) The PD gain achieved through optimal allocation of financial resources for maximizing future EPD within a cohort of 206 globally threatened birds. Dashed lines represent the standard deviation; circles represent downlisting events, following the ADEPD-cost ranking. The expected PD gain from current spending patterns is shown as a red line. The equivalent gain achieved by optimal spending of the same funds is shown in blue and is 3.8 times higher. The PD gain that would be achieved by downlisting all species and meeting the Aichi target is shown in green. (*b*) The rate of change of (*a*) to give a measure of PD gain per unit cost in relation to total expenditure, which varies from $0.059 to $3915 per year of PD saved.

The EPD in 50 years without conservation action is estimated to be 78 028 Myr (s.d. = 3663). The Aichi target obtained from improving the conservation status of all 206 species by one Red List category in 10 years corresponds to a gain of 385 Myr of EPD (s.d. = 24.75) (figure 3*a*). The total conservation expenditure required to meet this target for the 206 species in our study was US $3871 million (£2323 million) (figure 3*a*). By contrast, recent expenditure on the conservation of the same cohort over the past 10 years was only US $810 million (£486 million) (figure 3*a*). This expenditure is estimated to conserve only an additional 85.9 Myr of EPD in 10 years, representing only a quarter of that which would be saved if the Aichi target were met. Optimal spending of the same funds would be sufficient to save 340 Myr of PD, 3.9 times more than under the current spending allocation, and enough to save 88.4% of the PD gain from the Aichi target.

In an optimal spending scenario based on ADEPD-cost, an expenditure of US $1 represents sufficient funding to conserve as much as 16.69 years of PD (27.82 years of PD per £1 spent at current exchange rates). In a worst-case spending scenario, an expenditure of US $3914 (£2348) will conserve 1 year of PD (figure 3*b*). This makes $1 worth approximately 2.24 hours of PD under the worst-case spending scenario.

## 4. Discussion

### (a) Variation in phylogeny-based rankings

The ADEPD approach provides a fairer assessment of phylogenetically-based conservation prioritization by using extinction risk data for the entire tree under consideration and by incorporating the effects of downlisting on future EPD. This in turn makes ADEPD more compatible with available financial data for further analysis. While the HEDGE protocol includes the risk of extinction for all related taxa, it assesses the benefits of conservation effort that completely saves a species, which may require considerably more resources for species in higher threat categories. These methodologies are all distinct from more traditional prioritization methods, such as reserve selection algorithms, which are concerned with saving the greatest total number of species regardless of whether they are globally threatened or related to other species. Integrating a probability of extinction of a species within a given timeframe, and including phylogenetic data, can be more informative for minimizing biodiversity loss [38].

Although EDGE scores are less sensitive than HEDGE and ADEPD scores to different transformations of IUCN Red List categories to extinction probability [16], this is because the EDGE score for a species is only affected by the extinction risk of that species, whilst HEDGE and ADEPD are influenced by the extinction risk associated with every species in the tree [16]. We argue that it is better to use all available data and measure uncertainty (as ADEPD does) than ignore uncertain information simply to increase robustness.

Different transformations have different effects on the conservation metrics. For instance, using the IUCN 500 transformation for priority-setting is likely to result in overestimating the relative benefit of downlisting species from Endangered to Vulnerable, while downlisting species from Critically Endangered to Endangered would be considered less beneficial as the species will be likely to go extinct anyway within the 500 year time window in the absence of intensive intervention. This contrasts with the more optimistic opposing argument for assigning greater urgency of conservation support to more threatened species [39]: if we expect to be able to save most species from extinction or want to increase our chance of saving everything (rather than to maximize our expected gain in PD), then we should perhaps start with those species closest to extinction, an attitude that could be captured by a different transformation or by a more in-depth risk-based analysis that is still grounded on an ADEPD approach. In our analysis, we were concerned with maximizing conservation effectiveness in the relative short-term (within 50 years), and so the IUCN 50 transformation was chosen as most appropriate. Future work on this topic could conduct a thorough sensitivity analysis on these probabilities of extinction, which would help to evaluate the suitability of the various transformations for addressing different conservation prioritization questions. The appropriateness of estimating extinction risks from species' IUCN status can also be problematic. For instance, the IUCN methodology may underestimate the probabilities of extinction for certain species by overlooking potentially contributing threat factors, such as the impact of large-scale fragmentation on recolonization following adverse events [40]. Metapopulation models in Amazonian birds have highlighted the need to include this metric when categorizing threat risk, with 28 out of 58 species considered requiring re-assessment given their low metapopulation capacity [41]. Developing increasingly meaningful estimations on probabilities of extinction may therefore provide more informative inferences for species prioritization.

All PD-based prioritization approaches are sensitive to uncertainty over species threat status and phylogenetic placement [11,13,15,17,30,42]. The former of these has been highlighted as the main source of uncertainty in EDGE rankings [11]. Although we have addressed one major source of threat status uncertainty here by reassigning Data Deficient bird species to the Near Threatened category for the purposes of PD-based prioritization [13], other sources of uncertainty in prioritization rankings associated with threat status (e.g. status reassessments [43], variation in quantity and quality of available data for making assessments [44,45]) are harder to control for, but could be incorporated in the transformations. For example, the probability of extinction for an Endangered species could take into account the probability that the species is in fact Critically Endangered or Vulnerable and has been miscategorized as Endangered owing to uncertainty. In the avian phylogenies used for our analysis, all polytomies were resolved using taxonomic data prior to the final stage of tree construction, eliminating this type of uncertainty from calculations of evolutionary distinctiveness and instead encompassing it in a distribution of 10 000 different trees. However, polytomies are likely to be present in other large-scale phylogenies that we may also wish to assess using the ADEPD approach. An understanding of the impact of different polytomy resolvers on conservation prioritization, including with ADEPD, is therefore a potentially important area for future research.

Further differences between ADEPD rank and EDGE rank [13] are driven by consideration of the status of other species in the ADEPD priority-setting process; variability in confidence of these rankings, reflecting uncertainty in the phylogenetic placement of different species, provides greater uncertainty in ADEPD compared with EDGE. EDGE scores are expected to be less sensitive to uncertainty in phylogenetic placement than ADEPD scores because of the robustness of the ED metric [16]. Uncertainty is higher in edges where speciation intervals are shorter, which results in less evolutionarily distinctive species [37]; highly evolutionarily distinctive species may therefore be more likely to be placed correctly in a phylogeny and thus have a more certain conservation priority rank irrespective of which PD-based metric is used. Uncertainties in phylogenetic placement of deeper branches are, however, common in trees where fast speciation rates are followed by long periods of stasis in diversification that result in ‘bushes' in the phylogenetic tree [46]. As ADEPD is concerned with whole-tree PD, these bushes may account for variation within a species' ADEPD score. There is also geographical bias in the phylogenetic information available, with species in areas of Africa, Southeast Asia and Australia having higher uncertainty in their ED scores as a result [13]. This geographical asymmetry affects all phylogeny-based conservation approaches including EDGE, HEDGE and ADEPD, although it has not been explicitly tested in these metrics except for EDGE [13]. While the challenges of uncertainty are ubiquitous to assessments of future extinction risk, and especially to phylogenetically informed approaches, we must still attempt to seek ways to allocate resources. This means using more available data where possible and being open about uncertainty rather than ignoring useful information. ADEPD follows this philosophy and has many similarities with HEDGE; crucially, however, ADEPD has enabled us to incorporate new financial data into the analysis. Another factor that distinguishes ADEPD and HEDGE from EDGE is the potential to account for complementarity, or the changes in extinction probabilities that arise during species prioritization owing to the prior downlisting of more highly ranked species [16]. As the ranking differs between trees, we averaged the complementary ADEPD scores across all 10 000 trees. This approach may be further refined in future to account for the variation in complementarity across a large number of different phylogenetic templates; however, this will prevent full parallelization of the computational costs, which represents a significant barrier to tractability. We found that taking complementarity into account did not in fact make a difference to our overall ranking; this was most probably because there were not many examples of closely related species in our cohort under consideration. These issues could, however, become more critical in future studies with bigger sample sizes.

To date, all prioritization methodologies share a common limitation. In the case when two or more species have the same phylogenetic value, which of them should be given higher priority over other equally valuable and threatened species? This problem is common in EDGE, for instance in the amphibian priority list, where polytomic nodes of equally threatened species feature the same EDGE ranking [14]. Equally threatened sister species can have the same phylogenetic value even when phylogenies are fully resolved, whether based on EDGE or ADEPD scores, as observed in this study (e.g. the example of *R. pachyrhyncha* and *R. terrisi* described above). In this study, we demonstrated for the first time to our knowledge, the use of financial data as a tie-breaker in such situations. However, under a conceivable scenario where the phylogenetic value, extinction risk and financial requirements for the conservation of two or more species are all considered to be the same, other criteria such as cultural value (e.g. medicinal value [47]) or ecological value (e.g. ecological networks [48]) could also be taken into account to assist in prioritization.

### (b) Setting a price to phylogenetic diversity

The top-ranked ADEPD-cost species, which represents the highest-priority species to conserve in terms of amount of evolutionary history safeguarded per unit cost, is the Endangered Botha's lark, a species that sadly has received no targeted resources for species-specific conservation over the past decade [2]. It is interesting to note, however, that Botha's lark is not a particularly evolutionarily distinct species within the cohort of our analysis (ranked 103 in ADEPD rank and 132 in EDGE rank; ADEPD score = 1.184) and its top ranking is due to the low cost of downlisting the species, making it a ‘low-hanging fruit’ for PD-based conservation. If conservation management at a global scale was focused on conserving PD most effectively then it would be useful for activities to target such ‘low-hanging fruit’, i.e. those species that are expected to return the largest increase to future EPD per unit cost if downlisted. After such species have been downlisted, further conservation work leads to diminishing returns in conserving PD (figure 3). By contrast, the priority of the giant ibis, the most evolutionarily distinctive species in both our cohort and the EDGE bird list, is slightly offset in the ADEPD-cost ranking because of the much higher predicted expenditure considered necessary to downlist the species [2], although it remains a high priority overall even within the ADEPD-cost ranking (table 2). Perhaps a more reasonable compromise for a top ADEPD-cost species is represented by the Endangered tooth-billed pigeon (*Didunculus strigirostris*). This species has high ED and consequently is a top-ranking EDGE bird species [13], but the cost of downlisting it is also thought to be reasonably low [2], and so it is also ranked second overall in the ADEPD-cost ranking. Including a costing component to PD-based prioritization therefore provides a different priority list compared to EDGE and other metrics that only make use of data on phylogeny and threat. The differences shown by our approach from these metrics reflect the urgent need to find a consensus among conservation protocols for efficient conservation planning [18], and also the value of conducting comparative studies and cost–benefit analyses to explicitly and quantitatively compare different conservation strategies and forecast potential outcomes before the implementation of conservation initiatives [49]. For example, as our study compared the ability of two phylogeny-based prioritization methods to conserve future PD, one interesting direction for future research would be to compare these to a random species selection [50].

The recent expenditure of conservation resources for our cohort of bird species was shown to perform strikingly worse than the optimal conservation protocol for maximal PD gain with the same total available funds. This emphasizes the need for effective, empirical evaluation of conservation project investment and success using economic frameworks [51]. We show that whether recent conservation efforts are maintained, they are likely to achieve less than a quarter of the Aichi target of improving the status of all species in this cohort [32]. Failure to meet or even get close to this target reflects the limited amount of resources available for conservation, and also the poor distribution of those resources. In fact, our analysis illustrates that the amount of money spent on our cohort of 206 bird species over the last decade should be sufficient to downlist 136 species recognized as the highest priorities for cost-effective PD conservation in our ADEPD framework, enabling preservation of levels of future PD to reach 88% of that associated with the Aichi-like target outlined in our analysis. While there is a question over what percentage of resources should be used to allocate the remaining resources optimally, we have shown that there is a very large difference between optimal and current spending patterns and therefore that it may be worth making some effort to reassess avian conservation activities in order to move towards the optimal.

We do not advocate the removal of existing financial support for any species that are not ranked as high priorities in our return-on-investment analysis, as this may increase their extinction risk and thus decrease the quantity of PD likely to be preserved. Instead, we propose a complementary method for optimizing future resource allocation for bird conservation to maximize future EPD. Further financial considerations that may be harder to estimate for this sort of prioritization approach are also likely to affect the ultimate success of conservation efforts, providing a note of caution for our recommendations. The required expenditures used in this analysis are midpoint values derived from distribution estimates, and we did not address uncertainty in these values in our analysis. We recognize that some suggested expenditures may be underestimated, such as those required for pelagic birds, where estimates only considered protection of breeding colonies but not threats at sea such as fisheries by-catch [52]. The majority of estimated costs are also for site protection and other *in situ* conservation actions, with very few addressing further costs for potentially necessary intensive species-specific *ex situ* actions such as captive breeding [2]. Further ‘hidden’ costs may also be required if newly downlisted species still remain conservation-dependent in the long-term and thus require ongoing financial investment [53], and conservation action may also require much higher funds in future decades owing to emergent threats such as predicted impacts of climate change [54]. Conversely, costs for species conservation were considered separately, and we were not able to take into account potential shared costs for sympatrically occurring species [2]. In this analysis, we assumed the costs of downlisting were related to the improvement of a species' conservation status by no more than one IUCN category. The available estimated costs were for at least one category [2], so some species might be downlisted by more than one IUCN category with the same expenditure. As a result, it is possible that the ADEPD-cost scores of those species were underestimated in this analysis. However, we consider it unlikely that the minimum funds necessary to downlist a species once are in fact also sufficient to downlist it twice.

The cohort of 206 species used in this study was selected from the analysis of global financial efforts for bird conservation in reference [2]. Data for the initial cohort of 210 species selected in this study, from which the wider conservation costs for all bird species were estimated, were obtained through sending questionnaires to experts on all threatened birds. This cohort therefore represents those species for which data were received, and thus there is a further risk of geographical, taxonomic and/or other biases in the data available for our study, associated with variation in non-response from experts and/or lack of knowledge on estimated costs of downlisting or expenditure over the last 10 years. It should be possible to address this potential concern in the future as more extensive datasets on conservation costs become available. However, although financial data for additional bird species would lengthen our existing ADEPD-cost list, it would not change the current prioritization order of the existing cohort that we have already analysed.

## 5. Conclusion

PD is only one of many components of biodiversity, such as species richness, feature diversity and intrinsic value, which may not be closely related across wider species groupings, habitats or geographical regions that are targeted for conservation [5]. Other philosophical issues raised by conservation prioritization on the basis of PD, for example the relative value of conserving an evolutionary process rather than an evolutionary pattern [18] or the possible future evolutionary potential of species with few close living relatives [55], are also important to consider in future approaches. If PD is to be incorporated into wide-scale priority-setting, however, as we and many other conservation researchers advocate, it is important to assess the performance of PD-based metrics within a return-on-investment analysis. Only by using such an evidence-based economic approach, which is increasingly recognized as crucial for evaluating effectiveness across the wider conservation landscape [47,56,57], will it be possible to identify the most cost-effective ways to achieve optimal conservation of evolutionary history.

In this study, we conducted a broad-scale global prioritization analysis. However, we recognize that conservation decision-making, prioritization and resource allocation may vary when implemented at the regional or national scale, the levels at which many conservation programmes typically operate and at which resources are often made available. In practical terms, whereas we identify many non-charismatic species such as Botha's lark as top conservation priorities on the basis of PD-based return-on-investment analysis, it is also important to accept that conservation resources are likely to remain more readily available for ‘charismatic’ species; for example, 75% of the 16 bird species saved from extinction by conservation efforts are considered to be charismatic [58]. Indeed, it might have been difficult to raise the same funds for conservation if they were to be used on the best-value-for-money species instead of the more charismatic (and potentially more expensive) ones currently being protected.

Despite these caveats, however, our study offers initial insights into how financial resources may be better allocated in order to enhance PD-based prioritization and global biodiversity conservation in the coming decades. We anticipate that similar analyses could be expanded in the future to identify optimal resource allocation across the wider avian tree, or possibly across further smaller clades where complete data can be obtained. Similarly, future analyses could focus on other higher-order clades or geographical areas as appropriate data become available. We have outlined a strategy that has the potential to identify quantitative targets for conservation of PD under flexible financial and threat risk scenarios, and we are able to present unique and surprising results placing a value on the cost of saving a unit of PD across a substantial cohort of threatened bird species. The differences in benefit that we identify between current, optimal and worst-case spending scenarios are dramatic, ranging from over 16.7 years to less than 2.2 hours of PD saved per US dollar invested. This work highlights more generally the serious danger of ignoring economics when allocating precious and limited funds for conservation programmes.

## Supplementary Material

PD-based prioritisation and financial data for threatened bird species
