# Supplementary material for: The price of conserving avian phylogenetic diversity: a global prioritization approach
Source: Philos Trans R Soc Lond B Biol Sci. 2015 Feb 19;370(1662):20140004. doi: 10.1098/rstb.2014.0004 (PMC4290418; doi:10.1098/rstb.2014.0004)
Supplement: PD-based prioritisation and financial data for threatened bird species [file rstb20140004supp1.pdf]

Threat status, ADEPD scores (not taking into account complementarity), EDGE scores and estimated required and recent expenditure for the conservation of 206 globally threatened bird species.

Common Name	Scientific Name	IUCN Bird Red List 2013	ADEPD score	EDGE score	Required Expenditure (US$)	Recent Expenditure (US$)
Rufous-headed Hornbill	Aceros waldeni	CR	4.01935	5.27014	11700000	227000
Kiritimati Reed-warbler	Acrocephalus aequinoctialis	EN	0.22537	3.16048	2460000	75000
TahitiReed-warbler	Acrocephalus caffer	EN	0.27332	3.11996	1450000	3400
Millerbird	Acrocephalus familiaris	CR	0.72116	3.98234	2700000	1320000
Nightingale Reed-warbler	Acrocephalus luscinius	CR	1.92455	4.52984	88200000	3260000
Aquatic Warbler	Acrocephalus paludicola	VU	0.25316	3.44313	330000000	31900000
Purple-backed Sunbeam	Aglaeactis aliciae	EN	1.47184	3.98658	612000	9500
Raso Lark	Alauda razae	CR	1.6354	4.57759	514000	42000
Chestnut-bellied Hummingbird	Amazilia castaneiventris	EN	0.81087	3.55238	2140000	231000
Black-billed Amazon	Amazona agilis	VU	0.24148	3.04237	11400000	3240000
Tucuman Amazon	Amazona tucumana	VU	0.10574	2.85307	4020000	211000
Puerto Rican Amazon	Amazona vittata	CR	1.1709	4.12403	27500000	17200000
White-throated Grasswren	Amytornis woodwardi	VU	0.12183	3.76683	15000000	10800000
Ash-breasted Tit-tyrant	Anairetes alpinus	EN	1.21003	3.83951	8240000	992000
Auckland Islands Teal	Anas aucklandica	VU	0.02893	2.39253	5490000	0
Brown Teal	Anas chlorotis	EN	0.14459	3.08953	1200000	3230000
Hawaiian Duck	Anas wyvilliana	EN	0.56412	3.33374	9030000	260000
Amani Sunbird	Anthreptes pallidigaster	EN	1.69546	4.24002	29800000	16500000
Nilgiri Pipit	Anthus nilghiriensis	VU	0.16503	3.25948	4110000	10000
Araripe Manakin	Antilophia bokermanni	CR	1.01678	4.40679	12400000	1470000
Yellow-throated Apalis	Apalis flavigularis	EN	1.24639	3.86622	9520000	1000000
Taita Apalis	Apalis fuscigularis	CR	1.82498	4.56293	2930000	921000
Masafuera Rayadito	Aphrastura masafuerae	CR	1.99158	4.79684	4140000	86000
Southern Brown Kiwi	Apteryx australis	VU	0.22485	4.26348	2330000	1480000
Blue-throated Macaw	Ara glaucogularis	CR	2.94924	4.52755	1250000	1270000
Socorro Parakeet	Aratinga brevipes	EN	1.18366	3.58211	5600000	493000
Hainan Partridge	Arborophila ardens	VU	0.12538	3.12413	2710000	1440000
Great Indian Bustard	Ardeotis nigriceps	EN	1.26966	5.07401	11100000	1870000
Long-billed Tailorbird	Artisornis moreaui	CR	1.48871	4.70343	12300000	2260000

Threat status, ADEPD scores (not taking into account complementarity), EDGE scores and estimated required and recent expenditure for the conservation of 206 globally threatened bird species.

Pale-headed Brush-finch	Atlapetes pallidiceps	EN	0.97326	3.59634	775000	373000
Madagascar Pochard	Aythya innotata	CR	0.44433	3.85209	2740000	857000
White-bearded Antshrike	Biatas nigropectus	VU	0.18257	3.34327	662000000	16500000
Dwarf Olive Ibis	Bostrychia bocagei	CR	4.82401	5.52702	9440000	956000
Australasian Bittern	Botaurus poiciloptilus	EN	2.33523	4.70586	13000000	932000
Kittlitz's Murrelet	Brachyramphus brevirostris	CR	3.6651	5.05653	7800000	952000
Grey-cheeked Parakeet	Brotogeris pyrrhoptera	EN	1.17122	3.85902	10800000	104000
Usambara Eagle-owl	Bubo vosseleri	VU	0.21209	3.4447	13000000	16500000
Southern Ground-hornbill	Bucorvus cafer	VU	0.38222	4.38494	8250000	93000
Philippine Cockatoo	Cacatua haematuropygia	CR	4.66378	4.97623	2740000	870000
Yellow-crested Cockatoo	Cacatua sulphurea	CR	1.21083	4.60642	2550000	681000
Mangrove Finch	Camarhynchus heliobates	CR	0.3102	3.54036	5690000	973000
White-mantled Barbet	Capito hypoleucus	EN	0.16376	3.3464	61700000	141000
Bicknell's Thrush	Catharus bicknelli	VU	0.07072	2.94187	22500000	3320000
Gunnison Sage-grouse	Centrocercus minimus	EN	0.26027	3.63662	14100000	1290000
Esmeraldas Woodstar	Chaetocercus berlepschi	EN	0.16158	2.99619	31200000	12500000
St. HelenaP lover	Charadrius sanctaehelenae	CR	4.55037	5.42525	5150000	968000
Royal Cinclodes	Cinclodes aricomae	CR	1.05201	4.03896	7230000	872000
White-bellied Cinclodes	Cinclodes palliatus	CR	0.7931	3.96252	3020000	18000
Abbott's Starling	Cinnyricinclus femoralis	VU	0.33407	3.65362	22600000	1030000
Reunion Harrier	Circus maillardi	EN	1.65951	4.06398	101000000	86500000
Black Harrier	Circus maurus	VU	0.19589	3.40719	11200000	87000
Apolinar's Wren	Cistothorus apolinari	EN	1.27839	4.0311	1900000	23000
Golden White-eye	Cleptornis marchei	CR	1.93796	4.43167	41000000	16900000
Sangihe Shrike-thrush	Colluricincla sanghirensis	CR	3.48124	5.33329	1260000	815000
Nilgiri Wood-pigeon	Columba elphinstonii	VU	0.16896	4.74215	171000000	152000000
Mariana Crow	Corvus kubaryi	CR	1.21646	4.2988	29000000	12800000
Brown Eared-pheasant	Crossoptilon mantchuricum	VU	0.0046	2.74141	12600000	6290000
Malherbe's Parakeet	Cyanoramphus malherbi	CR	0.27379	3.91257	2940000	2340000
Okinawa Woodpecker	Dendrocopos noguchii	CR	0.85831	4.20686	49700000	3670000
Cebu Flowerpecker	Dicaeum quadricolor	CR	2.48594	4.92025	6300000	1010000
Tooth-billed Pigeon	Didunculus strigirostris	EN	9.75449	5.37124	695000	209000

Threat status, ADEPD scores (not taking into account complementarity), EDGE scores and estimated required and recent expenditure for the conservation of 206 globally threatened bird species.

Amsterdam Albatross	Diomedea amsterdamensis	CR	0.64077	4.8218	712000	350000
Chestnut-bellied Imperial-pigeon	Ducula brenchleyi	VU	0.19149	3.37688	10800000	1510000
Bicoloured Antvireo	Dysithamnus occidentalis	VU	0.16796	3.26237	9310000	2680000
Bahia Tapaculo	Eleoscytalopus psychopompus	CR	3.05993	5.26132	12100000	5490000
Colourful Puffleg	Eriocnemis mirabilis	CR	1.89478	4.55757	2930000	173000
Black-breasted Puffleg	Eriocnemis nigrivestis	CR	0.7985	4.40107	44000000	2380000
Chilean Woodstar	Eulidia yarrellii	EN	0.78714	3.30104	2750000	159000
Spoon-billed Sandpiper	Eurynorhynchus pygmeus	CR	10.79867	5.80416	26500000	1010000
Cerulean Paradise-flycatcher	Eutrichomyias rowleyi	CR	1.21228	4.27553	1260000	815000
Madagascar Serpent-eagle	Eutriorchis astur	EN	11.81134	5.54404	5420000	236000
Restinga Antwren	Formicivora littoralis	EN	1.01811	3.7339	7520000	207000
Djibouti Francolin	Francolinus ochropectus	CR	1.35209	4.37076	16900000	97000
Swierstra's Francolin	Francolinus swierstrai	EN	0.94203	3.6454	1140000	66000
Christmas Island Frigatebird	Fregata andrewsi	CR	7.88621	5.93714	1470000	244000
Polynesian Ground-dove	Gallicolumba erythroptera	CR	3.67077	5.11422	584000	126000
Negros Bleeding-heart	Gallicolumba keayi	CR	3.53034	5.0884	11000000	227000
Madagascar Snipe	Gallinago macrodactyla	VU	0.19859	3.50872	20700000	7780000
Okinawa Rail	Gallirallus okinawae	EN	1.4979	4.07452	26800000	5820000
Blue-crowned Laughingthrush	Garrulax courtoisi	CR	0.99975	4.19608	358000	67000
Collared Laughingthrush	Garrulax yersini	EN	0.72961	3.51651	1860000	783000
Northern Bald Ibis	Geronticus eremita	CR	2.94685	5.79345	12500000	3480000
Cloud-forest Pygmy-owl	Glaucidium nubicola	VU	0.19873	3.51228	17000000	59000
Cundinamarca Antpitta	Grallaria kaestneri	EN	1.8555	4.23066	3030000	45000
Jocotoco Antpitta	Grallaria ridgelyi	EN	1.85699	4.23822	5840000	1260000
Siberian Crane	Grus leucogeranus	CR	4.10309	5.16165	46800000	30400000
Black-necked Crane	Grus nigricollis	VU	0.05115	3.25615	16100000	7750000
California Condor	Gymnogyps californianus	CR	12.74715	6.31036	103000000	54000000
Crow Honeyeater	Gymnomyza aubryana	CR	2.47208	4.96298	29600000	10100000
White-rumped Vulture	Gyps bengalensis	CR	1.5315	4.6345	7640000	4340000
Indian Vulture	Gyps indicus	CR	0.4958	4.39003	7640000	4340000
Slender-billed Vulture	Gyps tenuirostris	CR	0.51012	4.39245	7640000	4340000
Black-cheeked Ant-tanager	Habia atrimaxillaris	EN	1.584	4.04402	1410000	0

Threat status, ADEPD scores (not taking into account complementarity), EDGE scores and estimated required and recent expenditure for the conservation of 206 globally threatened bird species.

Madagascar Fish-eagle	Haliaeetus vociferoides	CR	1.16291	5.01536	4090000	527000
Munchique Wood-wren	Henicorhina negreti	CR	1.82169	4.64574	4040000	114000
Forest Owlet	Heteroglaux blewitti	CR	13.29361	5.99484	4110000	39000
Liben Lark	Heteromirafra sidamoensis	CR	2.65268	4.72465	1110000	396000
Black Stilt	Himantopus novaezelandiae	CR	1.86359	5.08274	3630000	3280000
Bengal Florican	Houbaropsis bengalensis	CR	12.57593	6.00236	9700000	891000
Grand Comoro Flycatcher	Humblotia flavirostris	EN	1.34592	4.03283	1720000	514000
Montserrat Oriole	Icterus oberi	CR	1.08879	4.13744	2320000	26000
Gabela Bush-shrike	Laniarius amboimensis	EN	1.55805	4.05523	3600000	243000
Sao Tome Fiscal	Lanius newtoni	CR	1.67649	4.55747	9320000	338000
Black-billed Gull	Larus bulleri	EN	0.17846	3.05792	14800000	380000
Galapagos Rail	Laterallus spilonotus	VU	0.20157	3.48278	958000	8400
Junin Rail	Laterallus tuerosi	EN	1.55046	4.18472	3140000	58000
Sapphire-bellied Hummingbird	Lepidopyga lilliae	CR	0.43062	3.92895	2820000	54000
White-browed Tit-spinetail	Leptasthenura xenothorax	EN	0.76372	3.53703	2600000	872000
White-collared Kite	Leptodon forbesi	CR	3.39495	5.51539	1160000	58000
Grenada Dove	Leptotila wellsi	CR	2.63501	4.88534	14500000	1500000
Chinese Monal	Lophophorus lhuysii	VU	0.20795	3.54722	1050000	618000
Short-crested Coquette	Lophornis brachylophus	CR	1.25269	4.39627	6180000	155000
Akekee	Loxops caeruleirostris	CR	1.66335	4.279	29000000	2680000
Akepa	Loxops coccineus	EN	1.7262	3.58585	25600000	9360000
Zambian Barbet	Lybius chaplini	VU	0.14504	3.19499	47900000	8440000
Mountain Grackle	Macroagelaius subalaris	EN	0.93233	3.72914	940000	15000
Sharpe's Longclaw	Macronyx sharpei	EN	1.4696	4.10363	4670000	124000
Rufous-throated White-eye	Madanga ruficollis	EN	0.54385	4.23944	7810000	36000
Uluguru Bush-shrike	Malaconotus alius	CR	3.55066	5.13182	7040000	216000
Brazilian Merganser	Mergus octosetaceus	CR	0.83959	4.18225	56700000	5400000
Stresemann's Bristlefront	Merulaxis stresemanni	CR	2.89097	5.23536	8950000	917000
Perija Metaltail	Metallura iracunda	EN	0.71271	3.66876	3350000	39000
Socorro Mockingbird	Mimus graysoni	CR	1.34829	4.34803	4140000	493000
San Cristobal Mockingbird	Mimus melanotis	EN	0.1899	3.46966	1750000	15000
Floreana Mockingbird	Mimus trifasciatus	CR	1.53388	4.41128	16900000	113000

Threat status, ADEPD scores (not taking into account complementarity), EDGE scores and estimated required and recent expenditure for the conservation of 206 globally threatened bird species.

Amber Mountain Rock-thrush	Monticola erythronotus	EN	0.23178	3.5417	1130000	1850000
Puaiohi	Myadestes palmeri	CR	2.08106	4.70695	27800000	4590000
Paria Redstart	Myioborus pariae	EN	0.94016	3.4526	4930000	126000
Alagoas Antwren	Myrmotherula snowi	CR	2.41336	4.90171	3260000	824000
Cherry-throated Tanager	Nemosia rourei	CR	1.71707	4.79994	2930000	95000
Banded Ground-cuckoo	Neomorphus radiolosus	EN	2.71579	4.93039	8190000	451000
Sao Tome Grosbeak	Neospiza concolor	CR	1.75588	4.54925	9320000	962000
Fearful Owl	Nesasio solomonensis	VU	0.2073	3.58384	7610000	1320000
Red-tailed Newtonia	Newtonia fanovanae	VU	0.1864	3.50679	18900000	9800000
Javan Hawk-eagle	Nisaetus bartelsi	EN	0.71416	4.08565	861000	168000
Akikiki	Oreomystis bairdi	CR	3.29298	4.73369	29700000	2680000
Horned Guan	Oreophasis derbianus	EN	4.40718	4.67628	324000000	678000
Isabela Oriole	Oriolus isabellae	CR	0.1717	4.5367	4540000	365000
Anjouan Scops-owl	Otus capnodes	CR	1.8929	4.56824	14700000	4750000
Akohekohe	Palmeria dolei	CR	1.58144	4.38235	19900000	16400000
Forty-spotted Pardalote	Pardalotus quadragintus	EN	2.29284	4.71225	2940000	60000
White-winged Guan	Penelope albipennis	CR	0.7236	4.19277	3290000	1340000
Black Robin	Petroica traversi	EN	1.64789	4.53089	1270000	1170000
Stewart Island Shag	Phalacrocorax chalconotus	VU	0.05973	3.27313	224000	0
Chatham Islands Shag	Phalacrocorax onslowi	CR	2.45511	4.85362	470000	130000
Alagoas Foliage-gleaner	Philydor novaesi	CR	0.91616	4.09015	3260000	824000
Waved Albatross	Phoebastria irrorata	CR	3.61476	5.24648	4390000	478000
Urich's Tyrannulet	Phyllomyias urichi	EN	1.6564	4.16687	5500000	105000
White-necked Picathartes	Picathartes gymnocephalus	VU	0.33639	4.46713	4510000	77000
Trinidad Piping-guan	Pipile pipile	CR	0.13424	4.01481	4020000	60000
Philippine Eagle	Pithecophaga jefferyi	CR	13.47935	5.97685	21800000	3810000
Banded Wattle-eye	Platysteira laticincta	EN	1.81137	4.33114	4480000	2440000
Golden-naped Weaver	Ploceus aureonucha	EN	0.69844	3.44952	29400000	344000
Clarke's Weaver	Ploceus golandi	EN	0.7052	3.42594	12300000	3150000
Usambara Weaver	Ploceus nicolli	EN	0.68776	3.4456	41300000	1500000
Junin Grebe	Podiceps taczanowskii	CR	4.71685	5.51784	3060000	955000
Iquitos Gnatcatcher	Polioptila clementsi	CR	1.57802	4.50734	11200000	1150000

Threat status, ADEPD scores (not taking into account complementarity), EDGE scores and estimated required and recent expenditure for the conservation of 206 globally threatened bird species.

Palawan Peacock-pheasant	Polyplectron napoleonis	VU	0.27995	3.49173	16800000	13500000
Tahiti Monarch	Pomarea nigra	CR	2.21979	4.45989	2050000	365000
Fatuhiva Monarch	Pomarea whitneyi	CR	1.14114	4.20523	85400000	158000
White-throated Barbtail	Premnoplex tatei	VU	0.21513	3.51752	7660000	64000
White-shouldered Ibis	Pseudibis davisoni	CR	4.59328	5.62554	7920000	729000
Fiji Petrel	Pseudobulweria macgillivrayi	CR	2.86806	4.86407	2540000	334000
African Green Broadbill	Pseudocalyptomena graueri	VU	1.03286	4.53705	59900000	1740000
Maui Parrotbill	Pseudonestor xanthophrys	CR	2.2094	4.43584	22000000	16400000
Magenta Petrel	Pterodroma magentae	CR	1.86609	4.7048	4230000	531000
Galapagos Petrel	Pterodroma phaeopygia	CR	1.37065	4.63103	2810000	202000
Falcated Wren-babbler	Ptilocichla falcata	VU	0.06477	2.85344	13800000	1500000
Townsend's Shearwater	Puffinus auricularis	CR	2.50796	4.82297	7730000	679000
Balearic Shearwater	Puffinus mauretanicus	CR	2.02416	4.84906	6070000	735000
El Oro Parakeet	Pyrrhura orcesi	EN	0.62048	3.19681	2230000	2180000
Santa Marta Parakeet	Pyrrhura viridicata	EN	0.75098	3.25066	203000	36000
Jerdon's Courser	Rhinoptilus bitorquatus	CR	3.21989	5.32652	11800000	436000
Thick-billed Parrot	Rhynchopsitta pachyrhyncha	EN	3.04346	4.0526	1080000	23000
Maroon-fronted Parrot	Rhynchopsitta terrisi	EN	3.04346	4.0526	8180000	663000
Gough Bunting	Rowettia goughensis	CR	2.06562	4.58893	6890000	159000
Schouteden's Swift	Schoutedenapus schoutedeni	VU	0.17595	3.65988	34600000	3030000
Juan Fernandez Firecrown	Sephanoides fernandensis	CR	2.29092	5.13221	7140000	189000
Rubeho Akalat	Sheppardia aurantiithorax	EN	0.98988	4.05999	5520000	221000
Worthen's Sparrow	Spizella wortheni	EN	1.18321	3.83525	2150000	886000
Botha's Lark	Spizocorys fringillaris	EN	1.24794	3.96391	71000	0
Chinese Crested Tern	Sterna bernsteini	CR	1.35688	4.50324	3010000	561000
Kakapo	Strigops habroptila	CR	23.5638	6.28029	9380000	6930000
Blackish-headed Spinetail	Synallaxis tithys	EN	0.56272	3.32433	17500000	8150000
Craveri's Murrelet	Synthliboramphus craveri	VU	0.05085	3.28485	36500000	3250000
Mount Kupe Bush-shrike	Telophorus kupeensis	EN	2.047	4.3914	4550000	1430000
Seychelles Paradise-flycatcher	Terpsiphone corvina	CR	1.1359	4.29325	2570000	1190000
Giant Ibis	Thaumatibis gigantea	CR	31.89691	6.82656	6740000	157000
Niceforo's Wren	Thryothorus nicefori	CR	1.84019	4.63713	2180000	200000

Threat status, ADEPD scores (not taking into account complementarity), EDGE scores and estimated required and recent expenditure for the conservation of 206 globally threatened bird species.

Sombre Kingfisher	Todiramphus funebris	VU	0.14558	3.15831	7100000	6750000
Tuamotu Kingfisher	Todiramphus gambieri	CR	1.65795	4.54843	296000	444000
Marquesan Kingfisher	Todiramphus godeffroyi	CR	1.62218	4.52531	575000	725000
Pemba Green-pigeon	Treron pembaensis	VU	0.20587	3.48995	4010000	76000
Timor Green-pigeon	Treron psittaceus	EN	1.52224	4.09818	1450000	628000
Sao Tome Green-pigeon	Treron sanctithomae	VU	0.22339	3.51824	755000	755000
Santa Marta Wren	Troglodytes monticola	CR	1.54578	4.4932	859000	0
Taita Thrush	Turdus helleri	CR	2.75116	4.76912	5860000	1820000
Buff-breasted Buttonquail	Turnix olivii	EN	2.71274	4.6099	566000	43000
Sociable Lapwing	Vanellus gregarius	CR	3.92744	5.23294	1490000	644000
South Island Wren	Xenicus gilviventris	VU	1.28355	4.97076	2530000	248000
Spotted Ground-thrush	Zoothera guttata	EN	3.6457	4.58583	18000000	3230000
Mauritius Olive White-eye	Zosterops chloronothus	CR	0.60779	3.60925	2580000	642000
Bridled White-eye	Zosterops conspicillatus	EN	0.43041	2.99083	42000000	15900000
Sangihe White-eye	Zosterops nehrkorni	CR	0.5516	3.5993	1260000	815000
Rota Bridled White-eye	Zosterops rotensis	CR	0.60189	3.67204	25800000	15700000
Ranongga White-eye	Zosterops splendidus	VU	0.02898	2.10127	968000	0
